# A Further Step toward Sustainable Development: The Case of the Edible Mushroom Supply Chain

**DOI:** 10.3390/foods12183433

**Published:** 2023-09-15

**Authors:** Rachele De Cianni, Giovanna Cristina Varese, Teresina Mancuso

**Affiliations:** 1 Department of Agricultural, Forest and Food Sciences (DISAFA), University of Turin, Largo Paolo Braccini, 2, 10095 Grugliasco, Italy; teresina.mancuso@unito.it; 2 Department of Life Sciences and Systems Biology (DBIOS), University of Turin, Viale Pier Andrea Mattioli, 25, 10125 Torino, Italy; cristina.varese@unito.it

**Keywords:** mushroom, fungi, agri-food sector, supply chain, circular economy, Italy, Sustainable Developments Goals, multiple correspondence analysis

## Abstract

This study provides an accurate economic characterization of the supply of edible mushrooms throughout Italy within the European context to fill the relevant research gap and highlight barriers and opportunities that are consistent with the Sustainable Development Goals. Italian companies operating in this field were identified and economically characterized using the Chamber of Commerce’s Register of Companies. A qualitative web content analysis was then conducted to extract information about the marketed products, mushroom species, and retail channels, as well as the adopted certifications. The obtained data were quantitatively analyzed through descriptive statistics and multiple correspondence analysis. The Italian market is concentrated in northern areas of the country, and the limited company size indicates fragmentation at the production level, which led to Italy not being competitive enough and, thus, heavily rely on imports. Production is limited to less than 10 species, and innovative mushroom-based products, such as burgers, have shown a limited presence on the market, although they are gaining market share online. The novelty of growing kits highlights the potential to use food production waste to create fungal substrates. Investments in training new mushroom growers and studying new formulations and new fungal species are needed; these investments could allow greater market differentiation and be a good opportunity to promote local economies and create new job opportunities, thus meeting the requirements for sustainable development.

## 1. Introduction

In recent years, sustainable economic development has become a central theme of political and social dialogue at the global and local levels. The Farm to Fork Strategy of the European Green Deal aims to make food systems equitable, healthy, and environmentally friendly because they cannot withstand crises like the COVID-19 pandemic if they are not sustainable [1]. Even before the pandemic, the 2030 Agenda for Sustainable Development, which has been adopted by the member states of the United Nations (UN), was developed because of the urgent need for concrete action and cooperation between countries to end poverty, improve health and education, reduce inequality, combat climate change, and facilitate economic growth [2]. Several of the 17 Sustainable Development Goals (SDGs) are linked to the existing and potential benefits of mushroom production and consumption, but few publications have focused on these issues [3]. Considerations regarding the economic importance of the supply chain, as well as the excellent nutritional characteristics of mushrooms and their adaptability to the circular economy, render them suitable for sustainable development. They are very attractive as a food of which production and consumption, especially by European citizens, should increase in the future. As emphasized in the following paragraphs, mushroom production in Europe is currently rather limited, and this problem does not enable the supply chain to contribute to a great extent toward improving the environmental, social, and economic sustainability of society. Previous research carried out in European countries focused on finding strategies to increase mushroom consumption [3,4,5], but, to the best of the authors’ knowledge, an accurate and updated overview of the current European production and processing context is lacking in the scientific literature. Hence, the key contribution of this work is to provide an accurate economic characterization of Italian domestic mushroom production, which is necessary to enhance the supply chain. Italy was chosen as a case study in Europe since it is a country with good economic potential in the mushroom production sector, but its production is still limited, making it the perfect place to identify opportunities and barriers to the development of the mushroom sector, thus contributing to sustainable economic growth.

The remainder of this paper is structured as follows. The first part of the introduction is followed by three subsections: an in-depth discussion of the economic importance of edible mushroom production; a brief overview of the nutritional and medicinal properties of mushrooms; a description of the supply chain, with a particular focus on a novel means of production used by amateur growers—growing kits. Section 2 describes the methodology, and Section 3 discusses the main findings, while the main results are discussed in Section 4. Finally, the conclusions are given in Section 5. 

### 1.1. The Economic Importance of Edible Mushrooms

Nowadays, the edible mushroom market is showing an upward trend in terms of both production and consumption. FAOSTAT reported a growing trend in production from 2000 to 2020, with a production quantity of 42,792,893 tons in 2020 and a gross production value of USD 45,200,678 [6]. In Europe, the trend is similar but lower in terms of quantity, with a production value of 1,270,241 tons in 2020 [6]. The difference between the world’s production and European production is not surprising, considering that China alone is responsible for 93% of the world’s mushroom and truffle production, with 40 million tons produced in 2020 [6]. After China, Japan (471,810 tons) and the U.S.A. (370,280 tons) are the main producers, followed by the Netherlands (260,000 tons), India (201,000 tons), and Poland (182,900 tons). Among European producers, Italy appears in ninth place, with 69,210 tons produced in 2020. In Europe, the U.K. and Germany are the main importers, while Poland appears as the main exporter, followed by the Netherlands. There are about 16,000 known mushroom species throughout the world (considered to be macrofungi), including 3000 species considered to be totally edible, out of which only 100 are cultivated for economic purposes and 60 are cultivated for commercial purposes, while just over a dozen species are produced on an industrial scale in most countries. Of the 16,000 recognized species, around 700 are known to have medicinal properties, and about 500 are known to be poisonous [7,8]. Despite this wide variety, only five genera dominate the global market, and they represent 85% of the world’s total supply [9]. This list is headed by *Agaricus bisporus* (Button) and *Lentinula edodes* (Shiitake), which provide 38% and 22% of global production, respectively [9,10,11], as well as the highest market share, together with *Pleurotus* spp. (Oyster mushroom) [12,13]. In this context, SDG 8 includes the promotion of sustainable economic growth, which is possible, among other factors, through an increase in the diversification of production [2]; mushrooms could optimally fulfill this requirement, but the diversification of the species grown and consumed is still at its lowest potential.

### 1.2. The Nutritional and Medicinal Value of Edible Mushrooms

The aim of SDG 2 is to end hunger and improve global nutrition by ending all forms of malnutrition, while SDG 3 involves ensuring healthy living and promoting well-being at all ages. Mushrooms are able to optimally meet these goals. Indeed, they are low in calories and fat but have a good vitamin content [14,15,16] and high levels of phosphorus and iron [15,17], and they are considered to be a novel source of dietary fiber thanks to the presence of a cell wall that is rich in chitin, β-glucans, and mannans. These molecules are useful for strengthening the immune system, have anti-cancer functions [18], and can act as prebiotics [19]. In addition, the protein value of mushrooms varies from 18.87 to 36.96% of their dry weight [20], and their protein composition is particularly interesting compared to other plant-based proteins since they usually contain all essential amino acids (EAAs) needed to fulfil human dietary requirements. Thus, mushrooms are a food product with a high-quality protein profile and could, therefore, be a good functional food and a promising meat alternative [20,21,22,23] because of their particular texture and umami flavor [17,24]. Finally, mushrooms can be considered to be a cheap product that is more affordable for the poorer segments of the population compared, for example, to meat. In Italy, fresh cultivated mushrooms can cost the consumer EUR 5 to 10 per kilo at the supermarket, while, for example, a meat burger can cost EUR 15 to more than 20 per kilo [25,26].

### 1.3. Supply Chain Features

**Figure 1:**
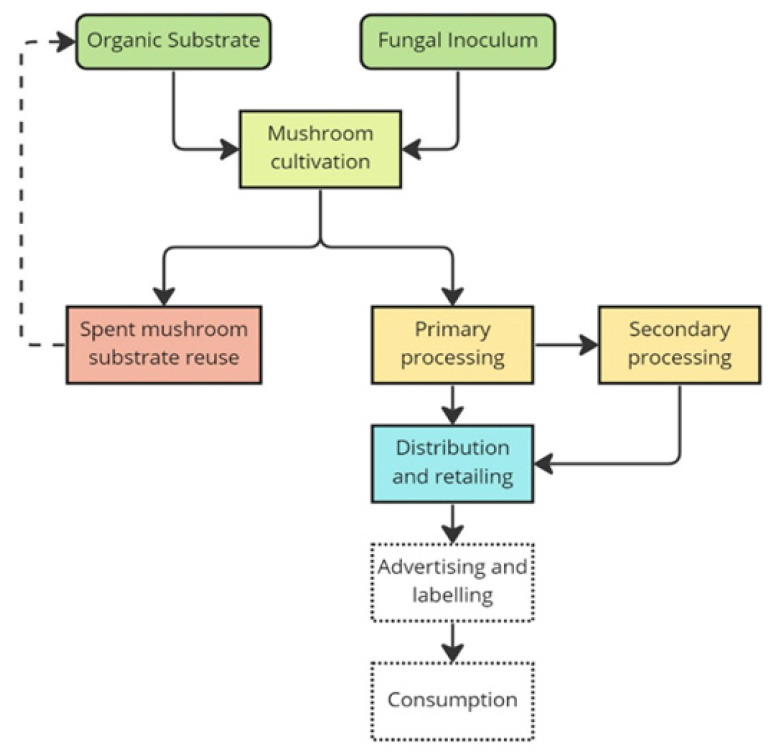
Edible mushroom supply chain. The colored boxes indicate the stages of the supply chain that were examined, while the dashed boxes indicate the stages that were not considered in the study because they were beyond the scope of this study.

Nowadays, mushroom cultivation can be simplified using relatively basic agricultural techniques. Moreover, producers can take full advantage of technological and industrial progress; however, to be efficient, such cultivation requires both scientific knowledge and practical experience [8]. Mushroom production consists of two steps: the production of growth substrates that are suitable for the individual fungal species, followed by the cultivation of the fungus. In this respect, companies can be defined as “closed-cycle mushroom farms” if they are involved in both production phases. A promising industrial approach used to reduce production costs is the solid-state fermentation (composting) of agro-industrial residues to produce vegetative mycelia, as well as the investigation of other unexplored micro-organisms, fermentation conditions, and potential substrates [22]. Indeed, mushrooms are able to grow on a variety of organic substrates, such as waste derived from wood, paper, and agricultural industries [8,20,27], thus perfectly fitting into the circular economy. In addition, once the mushroom cultivation cycle is completed, the remaining substrate is a lignocellulosic by-product called SMS (spent mushroom substrate), which can be reused as biofertilizer and soil amendment for biofuel or feed production or recycled as a substrate to begin a new mushroom cultivation cycle [28]. As a result of these characteristics, edible mushroom production is also able to satisfy SDG 12, which aims to achieve “sustainable management and efficient use of natural resources”, and SDG 13, which highlights the urgent need to combat climate change and its impacts. Currently, fresh products are dominant on the edible mushroom market, but there is increasing interest in processed products, especially in Western countries [17,29]. However, fresh mushrooms are highly perishable and deteriorate within a few days, and in some cases, even after a single day. To overcome this problem, one of the processes most commonly performed on fresh mushrooms is drying, which allows their shelf life to be greatly increased [30,31]. In addition, the increasing interest of consumers in products that are easy to prepare [32] has contributed, in recent years, to the emergence of several processed mushroom-based or mushroom-containing products [33,34,35]. Hence, the edible mushroom supply chain can be simplified, as shown in Figure 1.

#### Growing Kits

Growing kits are growth substrates obtained from food by-products that include fungal mycelium already inoculated to allow the amatorial cultivation of mushrooms [36], either indoors or outdoors, depending on the species grown. It is a fairly innovative venture that contributes to the circular economy, but, to the best of the authors’ knowledge, it has not yet been discussed in any great detail in any scientific publication. During the COVID-19 pandemic, the sales of mushroom growing kits increased by up to 400% compared to previous years, in part because people could not carry out their normal activities, as well as due to growing public interest in becoming more self-sufficient [37]. In Italy, companies that deal with the production of substrates for use in growing kits provide a small and useful guide to help customers attain better mushroom growth, which indicates a series of operations that should be performed, either inside the package or on top of the package itself, because consumers are generally uninformed about the optimal growing conditions required by a given species. 

### 1.4. Objectives and Research Questions

In an analysis of the supply chain, it is important to consider not only how consumption patterns influence food demand, but also to move in the opposite direction, that is, from supply to demand, with human health being the focal final point [38]. In this context, the main scope of the present study has been to characterize, in an economic dimension, the supply of edible mushrooms and identify and describe existing producing and processing companies in Italy, as it is a country with economic potential in this sector, with a focus on the early stages of the supply chain (which are colored in Figure 1). The study’s expected contribution is investigating the actual Italian production and processing context and using it as a case study to provide an overview of the main aspects and features of the supply of edible mushrooms. In doing so, it is possible to identify opportunities and barriers to allow the sustainable development and economic growth of the Italian edible mushroom market. Hence, this paper attempts to answer the following research questions:

- What is the economic capacity of companies that produce and/or process mushrooms in Italy in the European context?
- Which species are the most widely grown, and which products are the most frequently marketed?
- Which variables could be barriers to or opportunities for developing sustainable supply chain development?
- What are the peculiarities of growing kit production in Italy?

## 2. Materials and Methods

**Figure 2:**
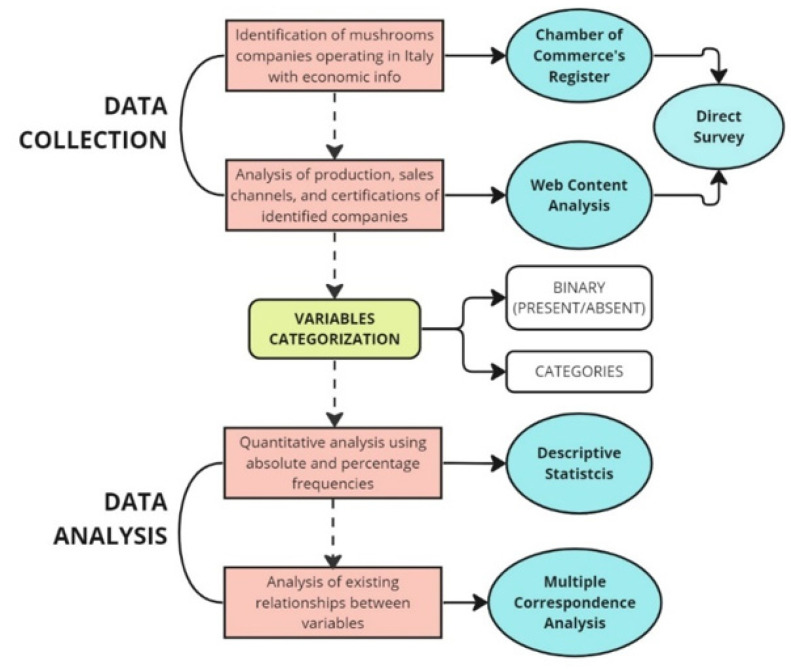
Outline of the stages of the research design followed in the present study.

To achieve our research objectives, a mixed qualitative and quantitative analysis was performed based on the subsequent steps. The research design is depicted in Figure 2. 

### 2.1. Data Collection

Initially, the companies involved in the production and/or processing of mushrooms operating on Italian territory were identified via the Chamber of Commerce’s Register of Companies [39]. This register can be described as the repository of information of all businesses, regardless of their legal structure or sector of economic activity, including their headquarters or local units on Italian territory; thus, it was deemed suitable for the purposes of the present study. Consequently, basic economic and descriptive information, such as the business name, geographical location, year of establishment, and, in some cases, the number of employees and turnover, was obtained. For the identified variables, being continuous or nominal, it was possible to assign a category to each company as follows:

- Geographic location: north, center, or south and islands.
- Business name: limited liability company, individual company, simple partnership, co-partnership, cooperative, joint-stock company, ordinary limited company, or limited company.
- Year of establishment: Before 1970 or after 1970. The year 1970 was chosen as the threshold value because it was the year in which the number of mushroom production-related firms began to rise in Italy.
- Turnover: The turnover categories were less than EUR 9 million and more than EUR 9 million. A total of EUR 9 million was chosen as the threshold value as it represents the possible turnover limit for a company to be defined as a “small enterprise” within the category of small- and medium-sized enterprises (SME), i.e., EUR 10 million.
- Number of employees: 1 to 10, 11 to 50, 51 to 250, or more than 250.

Hence, a database of Italian mushroom companies with basic economic variables was created. Afterwards, a qualitative web content analysis (WebCA) was performed using the identified individual companies’ websites to extrapolate relevant information, such as the main commercialized mushroom food products produced, the cultivated or processed fungal species, the retail channels, and the main product and process certifications possessed by the companies, following the first steps of the supply chain (Figure 1). WebCA is a technique that originated from the more common content analysis technique, the aim of which is to objectively and systematically describe a piece of web content [40] using corporate websites, which are one of the most widely used means of obtaining information about the products, history, and values identified by the company itself [41]. Qualitative data, such as information regarding commercialized mushroom food products, cultivated/processed mushroom species, retail channels, and the presence of product or process certifications, were categorized according to a binary scheme by identifying each variable as being present or absent for each firm and added to the database. Based on the information obtained via WebCA, the categories have been identified and grouped as follows:

- Mushroom food products: no processing, portioned, fresh (or not otherwise specified), in oil, dried, frozen (also breaded), pickled, sauces/soups, condiments (oil, spices), snacks/sweets, pasta/rice, coffee/tea, and burgers;
- Mushroom species: *Boletus edulis*, *Agaricus bisporus*, *Agaicus campestris*, *Pleurotus ostreatus*, *Pleurotus eryngii*, *Pleurotus cornucopiae*, *Tuber* spp., *Lentinula edodes*, and *Ganoderma lucidum*;
- Retail channels: large-scale retail (LSR), retail sale (including pharmacies and herbalists), firm’s own site, online (platforms), company stores, wholesale, and business-to-business (B2B) channels;
- Product or process certifications: ISO 9001, ISO 14001, ISO 22000, GRASP, IFS (International Featured Standard), BRC (British Retail Council), Global GAP, GMP, “Fungo Italiano Certificato”, SQNPI, and Organic.

All of the identified companies were sent a direct link to an online survey, which contained questions used to confirm the information found on the Chamber of Commerce’s Register of Companies and company websites, such as the business name, turnover, mushroom products marketed, mushroom species used, etc. These details were used to enrich the established database. In addition to these questions, other questions were added to obtain information that it was impossible to find online, which is presented in Section 3.3.

### 2.2. Data Analysis

The obtained data were then quantitatively analyzed by means of descriptive statistics using absolute and/or percentage frequencies, and they are presented as tables to simplify their interpretation. Since this paper is an exploratory study, an initial descriptive analysis of the obtained information was considered to be necessary for the purpose of this research. 

Then, a multiple correspondence analysis (MCA) was conducted with the aim of a deeper exploratory investigation and identifying useful suggestions for stakeholders. MCA is a multivariate statistics method that allows the relationships between more than two categorical variables to be analyzed [42]. Therefore, it can also be considered to be an extension of the correspondence analysis and a generalization of the principal component analysis when the variables that have to be analyzed are categorical rather than quantitative in nature [43,44]. MCA allows the number of variables to be reduced and the relationships between the variables to be deduced; dimensions are “extracted” to maximize the distances between points in a row or column [45,46]. The total inertia was considered to be representative if it reached at least 70% [47]. Variables were generally represented in a two-dimensional space called a biplot, which was used in MCA to graphically identify the relationships between variables. Variables were plotted as coordinates in the new factorial spaces, which comprised the dimensions obtained via the MCA [48]. The obtained biplot was presented to easily visualize the relationships between the variables. Since the MCA showed the sample profiles, including variables that were not essential to answering the research question, such as the year that the company was founded or its geographic location, it may interfere with the profile configuration [44]. For this reason, these variables were treated as supplementary variables in the analysis. In addition, to obtain further details for this study, an online survey was sent to all identified companies. This survey contained other specific questions regarding the main critical issues encountered by companies during production or processing and the criteria they adopted to choose species to cultivate or process, as well as questions regarding the marketed products, sales channels, and certifications used to confirm the information obtained via the websites or fill in any gaps. Unfortunately, only 22 companies filled in the questionnaire, and some of these responses were incomplete; therefore, only a qualitative discursive evaluation of the answers was conducted rather than quantitative analysis.

## 3. Results

### 3.1. Descriptive Statistics

**Figure 3:**
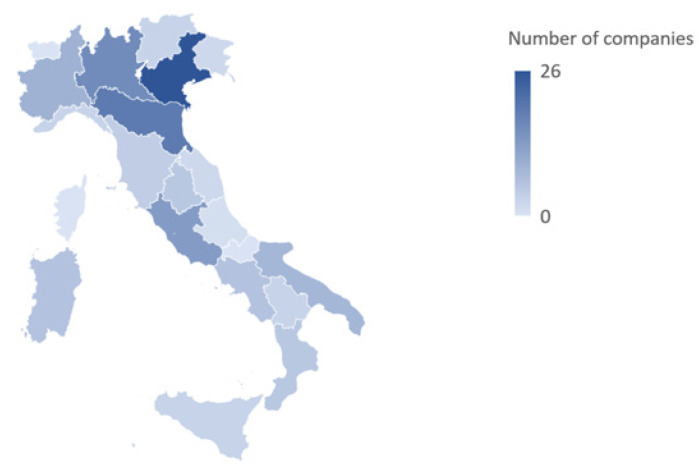
Distribution of edible mushroom cultivation and processing companies among different Italian regions.

The analysis identified 136 farms and firms in Italy that deal with mushroom cultivation and/or processing. The majority of these firms operate in the north (60.3%), followed by the south and islands (21.4%) and the center (18.3%), as shown in Figure 3; 82.5% of the companies were founded after 1970.

A large proportion of firms are limited liability companies (37.6%), followed by simple partnerships (21.8%) and individual companies (21.8%), while just a small proportion are co-partnerships (7.5%), cooperatives (4.5%), joint-stock companies (3.8%), ordinary limited partnerships (2.3%), and limited companies (0.8%). The north is home to the companies with the highest production capacity, such as cooperatives (66.7%), limited liability companies (52%), and joint-stock companies (50%). On the other hand, individual companies are more homogeneously distributed across the center (44.8%), north (34.5%), and south and islands (20%), as are simple partnerships, of which are 36.7% are found in the north, 30% are found in the center, and 33.3% are found in the south and islands. This division is also reflected in the turnover values and the number of employees of the companies: for 80% of the companies whose turnover could be surveyed (45), it was less than EUR 9 million, and the number of employees did not exceed 50 in 83.3% of the cases. 

The most widely marketed products are dried mushrooms and mushrooms in oil, followed by soups and sauces and fresh products, frozen mushrooms including breaded mushrooms, portioned mushrooms, condiments, pasta and/or rice pickles, burgers, snacks and desserts, coffee, and tea. There are just four mushroom farms that exclusively engage in cultivation without processing their products, three of which are located in Northern Italy.

In Table 1, the selected products are divided into minimally processed (fresh, dried, in oil, and portioned) and highly processed products (sauces/soup, frozen and breaded, condiments like oil and spices, pasta/rice, pickled, snacks/sweets, burgers, and coffee/tea). This division shows that about half of the identified companies are mainly engaged in cultivation and minimal processing, while the other half also produce processed products; unfortunately, eleven firms did not specify which product they cultivated or processed on their website, meaning that there are information gaps for these companies. 

The most frequently cultivated or processed fungal species, namely *Boletus edulis* (porcino), which is a fungus that is generally not cultivated and only collected by experienced gatherers [45], is instead used by 20% of the surveyed enterprises. This species is followed by *Agaricus bisporus* (16%), *Pleurotus ostreatus* (13%), and *Tuber* spp. and *Pleurotus eryngii* (12%). All of the other species are used much less frequently (Table 2). 

Table 3 shows the different numbers of the different mushroom species used in the production and commercialization of the identified mushroom products. *Boletus edulis*, *Agaricus* spp., and *Pleurotus* spp. are, once again, the most widely used species in all food products, and *Agaricus* spp. is the most frequently cultivated by farms that do not perform any kind of processing. 

*Tuber* spp. is also widely used, and interestingly, *Ganoderma lucidum*, which is known for its medicinal properties [49], is the only species used to enrich coffee or tea. Moreover, Lentinula edodes is widely processed by companies that produce burgers.

The majority of companies mainly sell their products in the wholesale market (55), and most of the other companies sell their products in the retail market (42). Interestingly, the third most used sales channel is the company’s own website, which is used by 34 companies and preferred to large-scale retail channels, which are used by 26 companies. Company stores (19), business-to-business transactions (9), and online sales (6) through platforms such as Amazon are the least used retail channels (Table 4). 

It was also possible to trace the presence of product or process certifications for 90 companies, and the most common certifications appeared to be the IFS (International Featured Standard) and Organic certifications, which 20 and 19 companies possessed, respectively. A large number of companies (11 and 10) also possessed the ISO 9001 certification for their quality management systems (International Organization for Standardization) and BRC (British Retail Council), while 9 companies possessed the “Fungo Italiano Certificato” (Italian Certified Mushroom). The aim of both IFS and BRC is to ensure high food safety standards throughout the production chain. Moreover, the “Fungo Italiano Certificato” attests that the fungi were cultivated in Italy via a unique food traceability system, mainly using physical and biological systems to perform pest and weed control in Italian soil composed of biodegradable and controlled organic raw materials, as well as a low energy and water consumption system [50]. Other certifications held include ISO 22000 for food safety system management, Global GAP (Good Agricultural Practices), SQNPI (National Quality System of Integrated Production), ISO 14001 for environmental management systems, and GRASP (Global Risk Assessment for Social Practices) (Table 4).

#### Growing Kits

WebCA identified eight Italian companies that produced growing kits (Table 5). Of the companies for which this information could be found (five), one firm started producing growing kits in 2010, one started in 2013, one started in 2016, one started in 2018, and one started in 2021. This development is, in fact, an extremely recent and innovative product. The average price is mainly between EUR 10 and 25 per kit, which includes a substrate mainly composed of straw (2), agro-food waste (1), wheat and molasses (1), and spent coffee grounds (5) that is packaged in cardboard and sold in a plastic bag (8). All identified companies prepare kits used to grow Pleurotus (8), and only a few prepare kits used to grow Agaricus (2). Online sales channels are frequently used by the companies (6 out of 8) to sell their kits. The other channels used are retail sales, including sales through the company space (6) or wholesale transactions (4).

### 3.2. Multiple Correspondence Analysis

An MCA was also performed to better explore the data. The presence of product or process certifications, turnover, the marketed products, the sales channels, and the fungal species used by companies were treated as the main active variables, while each company’s year of foundation and geographical location were treated as passive supplementary variables. The results showed that the two dimensions explained 83.75% of the total inertia, which, being more than 70%, was considered to be representative [47]. 

**Figure 4:**
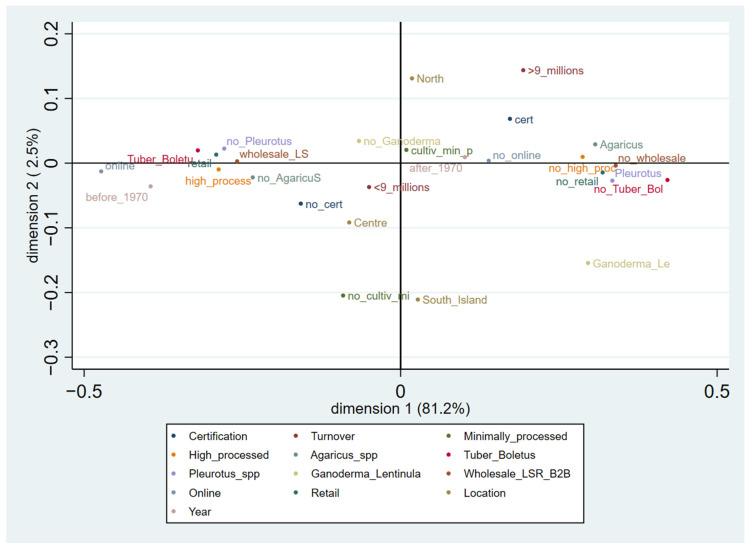
Multiple correspondence analysis (MCA) coordinate plot performed on the 136 identified Italian mushroom farms and firms. Variables listed in the legend may be present or absent. If absent, the variable name is preceded by “no” in the graph.

The resulting graph (Figure 4) visualizes the existing relationships between the variables, which can be read by considering their distance and position along the dimensions [43]. Negatively or positively correlated categories are located on opposite sides of the origin on the graph. In the first dimension, highly negative values are represented by companies that market highly processed products, prefer to use truffles (*Tuber* spp.) and porcini (*Boletus edulis*) over champignons or oyster mushrooms, and use both wholesale transactions and retail stores as their main retail channels. It is plausible to assume that some of these firms sell products online—probably the less common products—and were founded before 1970. The first dimension in the positive values instead points out companies that have completely different characteristics: they do not sell highly processed products and do not use retail stores to sell their mushroom products, which are mainly made of *Agaricus* spp.; it is plausible to assume that the majority of these firms have mainly dealt with cultivation or have used minimal processing since 1970, as well as that they do not sell their products online. The second dimension instead includes, in its negative values, companies that do not have any certifications (or whose presence could not be traced online) and have turnovers of less than EUR 9 million. On the opposite side of the second dimension, certified companies with turnovers of more than EUR 9 million appeared to be weakly correlated with companies that are involved in cultivation or use minimal processing. The presence of certifications and a turnover of over EUR 9 million appear to be correlated with the companies present in Northern Italy, where there are also some companies that are only involved in cultivation and carry out minimal processing of their products, while companies that are not certified and have lower turnovers appear to be correlated mainly with Central Italy and, to a lesser extent, the south and islands, where no cultivation or minimal processing of products takes place.

### 3.3. Direct Survey

A total of 136 Italian companies were sent an online survey to fill in, and 22 companies responded. In addition to the economic and production questions used to verify and implement the database described in Section 3.1 and Section 3.2, other questions were added to obtain details that could not be found online and will be discussed in this section.

It was revealed that the quantity demanded by the market is the most important parameter related to the preference for one species over another; this observation was emphasized by 68.2% of companies, followed by the cost of production and selling price (31.8%) and growing yield (31.8%). Ease of cultivation (18.2%), including the experience gained growing one species rather than another species, and the nutritional properties (4.5%) of the species were also considered to be important, albeit to a lesser extent. 

Moreover, the companies were asked which factors caused the greatest problems during cultivation, and a total of 13 companies answered this question. It was found that the control of parameters such as temperature and relative humidity (HR%) was the main difficulty; in fact, these parameters were considered to be important by 38.5% and 30.8% of the companies, respectively. Among the other critical factors, the maintenance of the cold chain during storage and transport, the presence of molds or pathogens that affect the substrate and/or the fungus, and the presence of insects, especially during the summer, obtained the same score (23.1% of companies). For those companies involved in cultivation, the incubation and harvesting stages were also viewed as critical (23.1%), while for those companies that produced semi-finished products or flours to be included in the final processed product, there were problems associated with the loss of raw materials and, therefore, waste generation (15.4%). Finally, the quantity demanded by the market, as well as the low selling price, were critical factors for 15.4% of companies. On the other hand, it was found that for those companies that were exclusively involved in mushroom processing, much of the raw materials processed were imported from abroad, i.e., half of the companies responding to question (8) said they imported materials from abroad, while the other half imported materials from either the Italian region in which the company was located or other regions.

## 4. Discussion

Italy has been confirmed as a country with good potential for the development of this sector at the European level, although its production and marketing have not yet reached the same levels as those of the major European producers, namely the Netherlands and Poland [6]. Indeed, Italian production and marketing were found to be concentrated in areas of Northern Italy, with the center and the south and the islands playing minor roles. This finding is not surprising considering that mushroom cultivation began earlier in the northern area, where companies with the most advanced production processes, facilities, and technology are located [17]. Moreover, the limited size of such companies indicates fragmentation at the production and processing levels, as this group is mainly controlled by companies with a limited production capacity, as is the case with limited liability companies, simple partnerships, and individual companies. On the basis of these considerations, it is possible to state that a concerted effort is still needed to enable small producers to grow and integrate within the market, thereby contributing to the achievement of the Sustainable Development Goals, particularly with regard to SDGs 2 and 9. In particular, the novelty of growing kits has pointed out that the use of food production waste to create fungal substrates is promising not only on a large scale, but also for amateur mushroom growers. Developing and increasing knowledge in this area would bring consumers closer to fungiculture and create new job opportunities, for example, thanks to the use of new species and new substrates in growing kits. 

However, the results show that the Italian market is growing but has still not reached sufficiently high levels to be competitive at the European level, and it still relies heavily on imports from foreign countries, especially with regard to the raw materials that have to be included in processed products. As noted by other studies, this issue exposes small producers to competition from imports of foreign agricultural products [51], which can be overcome with the creation of consortia or cooperatives to increase competitiveness [52].

As far as the diversification of marketed products is concerned, the analysis has shown that although most of the studied companies mainly perform minimal processing, new mushroom-based products are emerging in the market, such as mushroom-based burgers, snacks, and desserts, as well as tea and coffee enriched with mushrooms. The analysis of the species used for the production of the identified food products has revealed that there is not much diversification in Italian production. This result is in line with global statistics, according to which only five genera dominate the market [9]. Thus, the cultivation and processing of new species could help to develop the market and create new job opportunities not only in Italy, but also in other European countries. 

Moreover, mushrooms are a low-input crop that requires low investment, and they can be cultivated on a small scale using basic technology. Thus, the cultivation of mushrooms could increase female employment [17] and the employment of family farmers, thereby achieving productive employment and decent work for all, as targeted by SDG 8. 

Only 10 mushroom species are currently grown in Italy, which is a small number considering the 268 taxa that have been declared suitable for consumption in Europe, out of which 60 are cultivable and the rest are wild mushrooms [53]. Among these ten species, two prevail, namely *Agaricus bisporus*, which is the most cultivated mushroom in the world, and *Boletus edulis*, which is one of the mushrooms most valued by consumers but difficult to cultivate. This context underscores the importance of wild mushrooms in the edible mushroom sector and points to a gap at the legislative level. Indeed, in Europe, the different health and hygiene aspects, ranging from mushroom cultivation or harvesting and processing to the final purchase and the related control activities performed by the competent authorities, are based on general food safety laws and procedures (EC No. 178/2002), as well as regulations related to the hygiene of foodstuffs (EC No. 852/2004) and official controls (EU No. 2017/625). Furthermore, mushroom product labeling falls within the scope of EU Regulation No. 1169/2011 and further implementing regulations (EU No. 1308/2013 and EU No. 543/2011), which are responsible for outlining the marketing standards in the fresh fruit and vegetable sectors. However, implementing EU Regulation No. 543/2011 explicitly excludes non-cultivated mushrooms from its scope [54]. The EU regulatory framework has integrated previous national regulations that specifically address harvesting and marketing rules on both fresh and preserved epigean mushrooms to define the criteria required for the identification of, labeling of, and trade in edible species, but the specific regulations pertaining to European mushrooms are either guidelines or legislation based on traditional and mycological background, and they are managed by individual nations and, in some cases, such as in Italy, integrated with regional guidelines [54]. Hence, a gap exists at the EU level concerning the definition of specific measures to enable the safe commercialization of spontaneous mushrooms, considering the documented episodes of “false mycetism” that frequently occur because of the inadequate storage conditions implemented in the sales locations or the incorrect use conditions reported on the labels of both cultivated and wild mushrooms [55]. It is well known that mushrooms contain certain thermoresistant toxic compounds that can be responsible, for example, for chronic and acute gastrointestinal or neurological syndromes [54,56]. Thus, further European labeling harmonization would allow consumers to make a more informed and safer choice about mushroom products, thereby ensuring access to healthy and nutritious food for all, which is in line with SDG 2. Furthermore, better training of professionals and more control by local health associations are necessary to avoid the occurrence of these episodes, which could lead to reduced mushroom safety and cause fear of being poisoned among consumers, thereby discouraging and limiting their consumption. 

In addition, the correspondence analysis has shown that obtaining product and process certifications could be a benefit in terms of enhancing the supply chain and increasing competitiveness, especially for small mushroom enterprises, as pointed out in another study [57]. Indeed, the presence of certifications was found to be associated with a higher turnover among the enterprises. However, it should be pointed out that the presence of certifications and a higher turnover have also been associated with species that can be cultivated and the cultivation of which has a higher yield, such as champignon, but appear to be lower for companies that process wild species, such as porcini or truffles. In fact, the latter species seem to be more frequently used in the manufacturing of highly processed products that have a higher market value and have found a new market niche in online sales, including via company websites. As pointed out in another study, online marketplaces could help direct-to-consumer firms to compete for food clients on the Internet by reducing their search and transportation costs [58]. Online sales are also a promising sales channel, as most of the identified companies that produce mushrooms sell them through this channel. However, these data should be given proper weight, considering that the obtained information was mainly extrapolated from the analysis of the companies’ website contents, and it is plausible to assume that the online sales channel was more frequently “advertised” on websites and, thus, observed for more companies. This finding demonstrates the increasing importance of websites as corporate communication channels, as well as their importance, especially for companies that are less popular. Indeed, this analysis has brought to light the fact that for 11 out of the 136 identified companies, company websites did not show any of the marketed products, thus making it impossible for consumers to understand the activity carried out by the company. A similar argument can be made regarding product and process certifications, the presence of which should be promoted on company websites. Boosting the online marketplace to a greater extent would not only enable companies to increase their sales, but also promote the movement of goods between European countries by increasing their customer pools. However, the analysis also emphasized the increasing importance of certifications, with a particular emphasis on IFS certification (International Featured Standards) and organic certification. IFS certification demonstrates that the certified company has set up the appropriate processes to guarantee food and/or product safety, as well as that it has addressed and implemented customer specifications (https://www.ifs-certification.com/it/, accessed on 12 January 2023). Organic farming is an agricultural practice that seeks to produce food using natural substances and processes, thereby resulting in a limited environmental impact. To ensure a benefit for farmers, consumers should feel confident that organic production rules are respected [59]. Moreover, from a supply chain perspective, the adoption of certifications leads to an improvement in the transparency of operations, as well as the clearer distribution of liability among the supply chain agents and increased safety for consumers [60]. Hence, the presence of certifications, especially for cultivated mushrooms, could help to provide safer products to consumers and limit their fear of poisoning. Another strategy could be the creation of consumer cooperatives (co-ops) that provide education and direct services to consumers, granting them daily access to production information [61]. Furthermore, the growing relevance of online sales, especially those of highly processed “innovative” food products, has brought to light the potential of this new sales channel, which could provide a space for innovative products that are still struggling to gain a foothold in the market.

## 5. Conclusions

The present study sought to investigate the supply of edible mushrooms in Italy within the European context, with the aim of highlighting critical issues and barriers that must be overcome, as well as opportunities in the supply chain that would be useful for stakeholders. 

### 5.1. Practical and Theoretical Implications

To the best of our knowledge, no article has focused on the supply of mushrooms in Italy using both quantitative and qualitative analyses; the present study aimed to fill this research gap to allow the development of this sector. Other researchers could reproduce the methodological steps used in the present study to conduct a qualitative–quantitative examination of the supply of edible mushrooms in other countries and to assess other food products, examining either the same variables of interest or new variables that were not considered, such as marketing-related variables.

The results highlighted that success in this market segment will largely depend on the ability of entrepreneurs to identify the forces, tools, and methods that influence it, as well as their ability to integrate scientific expertise and market knowledge. In this context, growing kits appear to be a promising innovation, the use of which should be encouraged. In addition, fresh or minimally processed mushrooms, given their low price, stand out as products with excellent protein content that the less wealthy can afford, contributing to the well-being of society. Our results have shown that Italian production mainly involves fresh and processed products, while innovative products containing mushrooms, such as burgers or enriched products, still have very little space on the market, though they are gradually gaining importance, especially in online marketplaces. However, these new products are very interesting, especially as plant-based meat alternatives. They have high added value, and the use of certifications guaranteeing their food safety or organic production has proved to be a useful tool to perform market differentiation. It is, therefore, necessary to invest in new formulations and new fungal species to allow greater market differentiation and broaden market prospects. In addition, processed products suffer from fewer problems related to perishability, and there is less fear of poisoning from these products, which, generally speaking, are aspects that make mushrooms difficult to market and limit their consumption. Moreover, greater communication regarding the beneficial effects of mushrooms pertaining to health and clarity about the poisoning phenomenon (e.g., better training about conservation and cooking parameters for both food operators and consumers) would help to overcome these obstacles. In addition, ensuring higher process and product quality through certifications has proved to be an excellent way of increasing competitiveness. 

### 5.2. Policy Implications

Since fungiculture require hands-on expertise to grow certain species, economic investments by governments are needed to enable new generations to access and acquire the tools necessary to deepen this knowledge. As far as cultivated mushrooms are concerned, one of the major weaknesses is the high level of expertise required to cultivate a particular species; the control of the growth parameters, which can vary greatly from species to species; and the need to maintain the cold chain during the processing phases. These implications highlight the importance of funding and training a new generation of young mushroom growers who can successfully expand fungiculture, thereby enabling mushroom farms and firms to increase their competitiveness. However, some threats, which are related to the control of the growing parameters during cultivation and processing, as well as fluctuating market prices, remain. Thus, growers would benefit from financial help, or even better, specific training courses that could be sponsored by regional and agricultural services to overcome cultivation issues and allow the stakeholders to remain competitive. This observation could not only be true in Italy, but also in other European countries with similar production capacities, such as Germany, Ireland, Hungary, etc. [6]. However, mushrooms are a fairly cheap crop and quite technologically simple to grow as they require small areas and resources, can be grown anywhere all year round with low-cost starting materials, and have a low environmental impact, as well as very useful waste management results. For this reason, the cultivation of mushrooms could represent a real opportunity to promote local economies and create new job opportunities, thus meeting the requirements of sustainable development. 

In addition, greater harmonization at the European and Italian legislative levels, especially with regard to wild mushrooms, would make it easier for producers and collectors to access new markets and find new business opportunities, enabling the development of the supply chain and, therefore, limiting the poisoning phenomena. Finally, the success of the “Fungo italiano certificato” certification [49], which is held by many companies, is another means of differentiating between companies and could be taken as inspiration in other European countries or even for the creation of a Europe-wide certification.

### 5.3. Limitations and Future Research

This research suffers from certain limitations. Firstly, the difficulty of finding up-to-date data on the production and import and export of individual mushroom species has made data retrieval more complicated because the data are often presented in an aggregated form, i.e., mushrooms and truffles are presented together, meaning that some of the data may be approximations. Moreover, since most of the information used to perform the descriptive and multiple correspondence analyses was found on the companies’ websites and not via means of a direct survey, there may be errors due to incorrect or outdated information being present on the websites. Also, since the first part of the study is qualitative, it is not exactly replicable, because it could be subject to human error. However, the results indicate that there are many opportunities to perform future research, which is necessary to expand the knowledge framework concerning the edible mushroom market. 

Although this research offers an overview of the edible mushroom supply chain, the last stages of the supply chain, and in particular those of marketing, sales strategies, and consumption, have not been investigated in depth. For this reason, further research is needed to identify new marketing methods to increase trade and assess consumers’ attitudes and behaviors, which are crucial to developing this market. Research should also be undertaken to forecast consumers’ purchasing intentions, determine the variables that influence them to facilitate the promotion of edible mushrooms and their derived products, and increase demand for such products. There is also a need to find new ways of involving consumers in this category of products. If people are more familiar with a product and its characteristics, they appreciate it more and are more likely to become consumers. In addition, future research could perform a price analysis of the considered products and investigate consumers’ willingness to pay a premium price for emerging products containing mushrooms. Finally, the methodology of this research could be replicated in other European countries in order to define a European supply framework.

## Figures and Tables

**Table 1:** Firms that deal with the cultivation, minimal processing, or high processing of mushrooms in Italy (*n* = 136).

Company Features	Processing Firms (Sauces/Soups, Frozen, Condiments, Pasta/Rice, Pickles, Snacks/Sweets, Burgers, Coffee/Tea)		
**Cultivation and minimal** **processing firms** **(no processing, fresh, dried, in oil, and portioned)**	No	Yes	Total
No	11	0	11
Yes	63	62	125
Total	73	62	136

**Table 2:** Absolute frequencies and percentage frequencies of the companies that produce and/or process various fungal species in Italy, as revealed via analysis of their corporate websites.

Mushroom Species	Absolute Frequencies	Percentage Frequencies (%)
*Boletus edulis* *Agaricus bisporus* *Pleurotus ostreatus*	53	20.23
	41	15.65
	34	12.98
*Tuber* spp. *Pleurotus eryngii*	32	12.21
	31	11.83
*Pleurotus cornucopiae* *Lentinula edodes* *Agrocybe aegerita* *Ganoderma lucidum*	19	7.25
	20	7.63
	17	6.49
	10	3.82
*Agaricus campestris*	5	1.91

**Table 3:** The number of Italian growing and processing companies by species and products marketed on corporate websites.

	*Boletus edulis*	*Pleurotus* spp.	*Agaricus* spp.	*Tuber* spp.	*Lentinula edodes*	*Ganoderma lucidum*	Total
In oil	31	30	28	20	7	2	118
Dried	36	27	24	18	10	1	116
Sauces/soup	25	19	18	15	8	0	85
Fresh	21	18	19	13	5	1	77
Portioned	4	26	25	3	4	3	65
Frozen and breaded	22	13	11	10	9	0	65
Condiments (oil, spices, etc.)	15	14	13	8	6	1	57
Pasta/rice	13	10	7	7	4	1	42
Pickled	5	5	5	2	2	0	19
Snack/sweets	3	1	0	2	1	0	7
No processing	0	1	2	0	1	1	5
Burger	0	1	1	0	2	1	5
Coffee/tea	0	0	0	0	0	1	1
Total	175	165	153	98	59	12	

**Table 4:** Frequencies (absolute and percentage) of the companies involved in the mushroom supply chain based on retail channels and product and process certifications.

**Retail Channels**	**Absolute Frequencies**	**Percentage Frequencies (%)**
Wholesale	55	28.80
Retail sales	42	21.99
Company website	34	17.80
Large-scale retail	26	13.61
Company stores	19	9.95
B2B	9	4.71
Online (platforms)	6	3.14
**Certifications**		
IFS	20	21.74
Organic	19	20.65
ISO 9001	11	11.96
BRC	10	10.87
ISO 22000	9	9.78
Global GAP	9	4.35
“Fungo italiano certificato”	4	4.35
SQNPI	4	9.78
ISO 14001	3	3.26
GRASP	3	3.26

**Table 5:** The Italian companies that produce growing kits, with the year of production of the first kit, the price of the kit, the packaging materials, the first harvest of the mushrooms, the sales channels used, and the mushroom species. The “X” indicates that the company holds the feature specified in the column heading.

First Kit	Substrate	Price Per Unit (EUR)	Packaging Materials	First Harvest (Days)	Retail and Company Store	Online	Whole Sale	*Pleurotus*	*Agaricus*
2018	Straw and beetroot	10	Cardboard and plastic	30 days	X	X		X	X
2010	Straw	8.90	Cardboard and plastic	18 days	X	X	X	X	X
/	Wheat or millet, beetroot, and molasses	/	Cardboard and plastic	15–20 days	X		X	X	
2016	Coffee grounds	15	Cardboard and plastic	10–15 days		X		X	
2013	Coffee grounds	25.80	Cardboard and plastic	40 days	X	X		X	
/		/	Cardboard		X	X		X	
2021	Straw	10	Cardboard and plastic	20 days	X		X	X	
/	Straw	/			X	X	X	X	

## Data Availability

The data presented in this study are available on request from the corresponding author. The data are not publicly available due to privacy restriction.
